# Improved Induction of Anti-Melanoma T Cells by Adenovirus-5/3 Fiber Modification to Target Human DCs

**DOI:** 10.3390/vaccines6030042

**Published:** 2018-07-18

**Authors:** Dafni Chondronasiou, Tracy-Jane T. H. D. Eisden, Anita G. M. Stam, Qiana L. Matthews, Mert Icyuz, Erik Hooijberg, Igor Dmitriev, David T. Curiel, Tanja D. de Gruijl, Rieneke van de Ven

**Affiliations:** 1 Department of Medical Oncology, Amsterdam UMC, Vrije Universiteit Amsterdam, Cancer Center Amsterdam, 1081 HV Amsterdam, The Netherlands; dafni.chondronasiou@gmail.com (D.C.); t.eisden@vumc.nl (T.-J.T.H.D.E.); agm.stam@vumc.nl (A.G.M.S.); R.vandeven@vumc.nl (R.v.d.V.); 2 Microbiology Program, Department of Biological Sciences, College of Science, Technology, Engineering and Mathematics, Alabama State University (ASU), Montgomery, AL 36104, USA; qmatthews@alasu.edu; 3 Department of Genetics, University of Alabama in Birmingham (UAB), Birmingham, AL 35294, USA; icyuz@uab.edu; 4 Department of Pathology, Netherlands Cancer Institute/Antoni van Leeuwenhoek, 1066 CX Amsterdam, The Netherlands; e.hooijberg@nki.nl; 5 Division of Cancer Biology, Washington University, St. Louis, MO 63110, USA; idmitriev@radonc.wustl.edu (I.D.); DCuriel@radonc.wustl.edu (D.T.C.); 6 Department of Experimental Immunology, Amsterdam UMC, University of Amsterdam, Amsterdam Infection & Immunity Institute, 1105 AZ Amsterdam, The Netherlands

**Keywords:** melanoma, adenovirus (Ad)5/3, dendritic cell targeting, melanoma-specific T cells, MART-1, sentinel lymph node

## Abstract

To mount a strong anti-tumor immune response, non T cell inflamed (cold) tumors may require combination treatment encompassing vaccine strategies preceding checkpoint inhibition. In vivo targeted delivery of tumor-associated antigens (TAA) to dendritic cells (DCs), relying on the natural functions of primary DCs in situ, represents an attractive vaccination strategy. In this study we made use of a full-length MART-1 expressing C/B-chimeric adenoviral vector, consisting of the Ad5 capsid and the Ad3 knob (Ad5/3), which we previously showed to selectively transduce DCs in human skin and lymph nodes. Our data demonstrate that chimeric Ad5/3 vectors encoding TAA, and able to target human DCs in situ, can be used to efficiently induce expansion of functional tumor-specific CD8^+^ effector T cells, either from a naïve T cell pool or from previously primed T cells residing in the melanoma-draining sentinel lymph nodes (SLN). These data support the use of Ad3-knob containing viruses as vaccine vehicles for in vivo delivery. “Off-the-shelf” DC-targeted Ad vaccines encoding TAA could clearly benefit future immunotherapeutic approaches.

## 1. Introduction

The long-term survival rates for patients with metastatic malignant melanoma have dramatically increased over the past seven years with the effective clinical targeting of immune checkpoints like CTLA-4 and PD-1 [1,2,3]. With these successes, also the understanding arose of the importance of an inflammatory immune infiltrate in the tumor microenvironment for the clinical efficacy of these immunotherapies [4]. Patients who do not have a so-called “T cell inflamed/hot” tumor microenvironment, are less likely to respond to immune checkpoint blockade and may require a “priming therapy”, such as an anti-cancer vaccine, to kick-start the cancer immunity cycle [5].

Due to their unique ability to orchestrate T- and B-cell immunity, dendritic cells (DCs) are attractive targets for the delivery of vaccines. As professional antigen presenting cells (APCs) of the immune system, DCs are highly specialized in capturing, processing and presenting antigens to naive lymphocytes. For this purpose, DCs patrol the peripheral tissues to detect and take up antigens and, provided they receive sufficient maturation-inducing stimuli, then migrate into the paracortical areas of draining lymph nodes (LN) to engage and prime specific T cells.

The well-known and often used melanoma tumor-associated antigen (TAA), MART-1/Melan-A is an immunogenic self-antigen expressed in both normal and malignant melanocytes. While justifiably much of ongoing research focuses on patient-specific neo-epitopes as attractive antigens to target [6,7], over-expressed shared tumor antigens should not be disregarded as vaccine targets. In metastatic melanoma, it has been reported that the majority of tumor-infiltrating lymphocytes (TILs) derived from HLA-A2 positive patients were able to recognize the immunodominant epitope of MART-1, i.e., the fragment of the protein consisting of the amino acids 26–35 (MART-1_26–35_) [8]. Optimal delivery of TAAs to DCs is crucial for the enhancement of their therapeutic potential. Ex vivo modified autologous DCs have been used as melanoma vaccines to improve anti-tumor immunity [9,10]. However, laborious, expensive, and time-consuming ex vivo generation of DCs as well as their poor in vivo migration, often complicate their clinical utility and impair their efficacy [11,12]. An attractive alternative approach is based on the delivery of TAAs directly to tissue resident DCs in situ. In vivo targeting of DCs takes advantage of their intrinsic migratory and T cell stimulatory capacities. Numerous approaches have been designed for targeting DCs in vivo using vehicles such as monoclonal antibodies, nanoparticles, viruses, or liposomes in order to deliver the TAA of choice [13,14,15,16,17].

Adenoviruses (Ads) appear to be ideal vaccine vehicles as they can be genetically modified to stably carry large transgenes in their genome that remain episomal in the transduced host cells, minimizing the possibility of insertional mutagenesis [18]. Replication-deficient Ads can be easily propagated in high titers, enhancing their safety for in vivo use and simultaneously profiting of potentially immune-activating viral components that can function as vaccine adjuvant. Moreover, Ads can efficiently transduce both replicating and non-replicating cells [8] and induce robust humoral and cellular antitumor immune responses in vitro and in vivo [19,20,21]. Serotype-5 recombinant adenovirus (Ad5) encoding TAAs has been widely used as vehicle for gene transfer to human DCs [22]. As a member of the subgroup C adenoviruses, Ad5 binds to the cocksackie- and adenovirus receptor (CAR) as a primary docking receptor prior to infection, and uses integrin docking via hexon proteins for internalization. It has already been shown that recombinant Ad5 can act synergistically with maturation-inducing signals to generate highly immunostimulatory APCs [22]. However, DCs are relatively refractory to Ad5 transduction due to low levels or even lack of CAR expression on their cell surface [23]. In contrast, CAR is present at the surface of a wide variety of bystander cells [24]. To efficiently transduce DCs, Ad5 vectors need to be administered in vivo at high titers, which may also lead to the transduction of non-professional APCs and consequently result in sub-efficient T cell activation, T cell anergy or even induction of specific tolerance to the inserted transgene product [25]. Systemic use of Ad5 vector is also restrained due to a high prevalence of pre-existing immunity against this vector (in 50–90% of the population) [26]. To enhance the therapeutic potency and minimize unwanted side effects, new adenoviral vectors with broadened tropism are being developed [27] that allow redirecting of the adenovirus to target receptors that are highly, and selectively expressed on the surface of DCs.

Adenovirus-3 (Ad3) has been described to bind to the co-stimulatory molecules CD80 (B7.1) and CD86 (B7.2) on human monocyte-derived DCs (MoDCs) [28]. We previously analyzed a panel of different subgroup B/C chimeric and fiber-modified Ads, and compared them for their relative capacity to transduce human DCs [29]. The C/B-chimeric adenovirus serotype 5 (Ad5)/3, consisting of Ad5 fiber and Ad3 knob, was selected for its high-efficiency (skin) DC-transduction through binding to the B7-family members CD80/CD86 without interrupting subsequent T cell stimulation and proliferation. Ad5/3 was superior to Ad5 in the transduction of in vitro generated mature MoDCs, as well as the specific targeting of mature human myeloid DCs emigrated from skin (Langerhans cells and dermal DC) and residing in melanoma skin-draining lymph nodes (LN). Due to their more specific tropism, Ad3-based vectors may be less likely to infect non-professional APCs and cause undesirable side effects such as immune tolerance. The sentinel lymph node (SLN), as the first tumor-draining LN, is considered to be the most probable site of initial micrometastasis and is characterized by profound tumor-induced immune suppression. Also, the SLN is the primary site where naïve T cells are primed for the recognition of TAA by tumor antigen presenting, activated DCs. The generation of effective T cell mediated immunity in SLN is crucial for the elimination of metastases [30,31]. Whereas Ad5-based vaccines can often only be applied once, as an immediate induced or enhanced Ad-specific immune response will neutralize a booster vaccine [32], heterologous prime-boost vaccines incorporating Ad-based vaccines have been shown to be an appealing strategy for DC-targeted vaccination [33,34,35]. In this respect, vaccines based on alternative serotypes, such as the subgroup B1 adenovirus Ad3, with lower pre-existing immunity, might be particularly good candidates for such an approach.

In this study, DCs derived either from in vitro differentiated monocytes or ex vivo from melanoma-draining SLN were transduced with CD80/CD86-targeted fiber-modified Ad5/3 encoding full-length MART-1. Our main aim was to evaluate whether transduction of DCs by this chimeric, MART-1 expressing Ad5/3 could lead to the priming and activation of melanoma-specific CD8^+^ T effector cells. More importantly, we determined whether this vector could effectively target APCs in SLN of melanoma patients in order to enhance the frequencies of tumor-reactive effector CD8^+^ T cells. We compared the priming capacity of DCs transduced by the Ad5/3-MART-1 vector to DCs transduced by the Ad5-MART-1. Our findings suggest that an “off-the-shelf” DC-targeted Ad3-based vaccine could benefit future immunotherapeutic approaches by increasing tumor-antigen specific CD8^+^ effector cells both in tumor-draining SLNs and systemically, that could next be unleashed in their proliferation and killing capacity by immune checkpoint blockade.

## 2. Materials and Methods

### 2.1. Cell Lines

The melanoma cell lines Mel-AKR (MART-1^+^ HLA-A2^+^) and Mel-JKO (MART-1^+^ HLA-A2^−^) (Netherlands Cancer Institute, Amsterdam, The Netherlands) and the human glioblastoma cell line U251 (MART-1^−^ HLA-A2^+^) were cultured in Dulbecco’s Modified Eagle Medium (DMEM; Lonza, Verviers, Belgium) supplemented with 10% fetal calf serum (FCS; HyClone), 100 IU/mL sodium-penicillin, 100 μg/mL streptomycin, 2 mM l-glutamine, and 50 μM β-mercaptoethanol (2ME) (complete medium). The Epstein Bar virus (EBV)-transformed HLA-A2^+^ B-cell line JY was cultured in Iscove’s modified Dulbecco’s medium (IMDM; Lonza, Verviers, Belgium) complete medium. 

### 2.2. Monocyte-Derived DC Generation

Peripheral blood mononuclear cells (PBMCs) from HLA-A2^+^ healthy donors were isolated from buffy coats obtained from Sanquin blood supply foundation (Amsterdam, The Netherlands) using density gradient centrifugation over Ficoll as described in [36]. CD14^+^ monocytes were isolated from PBMCs by positive labeling using magnetic bead-conjugated anti-CD14 antibody (Miltenyi Biotec, Bergisch Gladbach, Germany) according to the manufacturer’s protocol. Isolated monocytes were cultured in IMDM complete medium supplemented with 1000 IU/mL recombinant human granulocyte colony stimulating factor (rhGM-CSF; Leukine, Berlex, Seattle, WA, USA) and 10 ng/mL interleukin-4 (IL-4; R&D systems, Abingdon, England, UK) over 5 to 6 days for the generation of immature DC. MoDCs were matured by culturing them for two more days in IMDM complete medium supplemented with a cocktail of maturation inducing cytokines: 10 ng/mL interleukin-6 (IL-6) (R&D Systems, Minneapolis, MN, USA), 1 μg/mL prostaglandin E2 (PGE2) (Sigma-Aldrich, Zwijndrecht, The Netherlands), and 25 ng/mL interleukin-1 beta (IL-1 beta) (Miltenyi Biotec), 2400 IU/mL tumor necrosis factor- alpha (TNFa) (Miltenyi Biotec).

### 2.3. Flow Cytometric Phenotypic Analyses

The MoDC phenotype was assessed using CD1a fluorescein-5-isothiocyanate (FITC)- and CD14 PerCP-labeled monoclonal antibodies (mAbs). To ascertain MoDC maturation, Phycoerythrin (PE)-labeled mAbs directed against human CD86 (BD Pharmingen, San Jose, CA, USA; 1:50) were used as well as isotype-matched control mAbs. Approximately 5 × 10^4^ cells were washed in phosphate-buffered saline (PBS) supplemented with 0.02% sodium azide (NaN_3_) and 0.1% bovine serum albumin (BSA), and incubated with the specific mAb for 30 min at 4 °C. 

PE-labeled HLA-A2 tetramers (Tm) presenting the MART-1_26–35L_ epitope were produced as described previously [37] and were used for cytofluorometric analysis of primed CD8^+^ T cells (generated as described below [38]). Tm staining was performed for 20 min at 37 °C, followed by incubation with FITC-labeled mAbs against CD8 (BD Biosciences, San Jose, CA, USA; 1:100). Dead cells were excluded by adding 0.5 μg/mL propidium iodide (ICN Biomedicals, Zoetermeer, The Netherlands). Stained cells were measured on a FACSCalibur^TM^ flow cytometer (BD Biosciences, Franklin Lakes, NJ, USA) and data were analyzed by the Cell QuestPro software.

### 2.4. Generation of Adenoviral Vectors

The Ad5-MART-1 vector was used and described previously [39]. To generate Ad5/3-MART-1, pShuttle-CMV-MART-1 was constructed and recombined with Ad5/3 backbone (pVK903K) plasmid. In order to generate pShuttle-CMV-MART-1, PCR was used to incorporate *Sal*I and *Not*I sites onto the MART-1 cDNA. The following primers were used: SalI Forward Mart-1: GCGAAGTCGACATGCCAAGAGAAGATGCT and NotI reverse-TGCTCGGCGGCCGCTTAAGGTGAATAAGGT. The resulting PCR product was digested with *Sal*I and NotI restriction enzymes, along with pShuttle-CMV plasmid. The MART-1 fragment was cloned into the pShuttle-CMV plasmid and the resultant pShuttle-CMV-MART-1 plasmid was sequenced for verification. pShuttle-CMV-MART-1 was linearized with *Pme*I restriction enzyme. The recombination of Ad5/3 backbone (pVK903K) plasmid and linearized pShuttle-CMV-MART-1 was performed in *Escherichia coli* BJ5183 (Stratagene, CA, USA), leading to the identification of positive vector clones by means of PCR and sequencing.

To rescue the vector, the recombinant adenoviral genome was digested with *Pac*I, and transfected into the Ad5-E1-expressing HEK293 cells using Lipofectamine 2000 Reagent (ThermoFisher Scientific, Waltham, MA, USA). Multi-step large-scale propagations of recombinant Ad5 vector were performed after the vector was rescued. Rescued viruses were purified by double cesium chloride ultracentrifugation and dialyzed against phosphate buffered saline without Mg^2+^ or Ca^2+^ and with 10% glycerol. Viruses were stored at −80 °C until use. To titrate the purified vectors, physical titers, expressed as viral particles (VPs) per mL were measured using absorbance at 260 nm. 

### 2.5. In Vitro Ad Transduction

Replication-deficient Ad5 and Ad5/3 viruses were used for MoDC transduction. These viruses were encoding either green fluorescent protein (GFP) or full length MART-1. In vitro-generated mature MoDC (1 × 10^5^) were transduced with the adenoviruses at a multiplicity of infection (MOI) of 1000 viral particles (VP). In short, mature MoDCs were resuspended in 500 μL serum free IMDM supplemented with 100 IU/mL sodium-penicillin, 100 μg/mL streptomycin, 2 mM l-glutamine and 50 μM β-mercaptoethanol (2ME). The indicated Ad were then added to the cells and incubated at 37 °C and 5% CO_2_. In two out of four experiments, Ad-5-MART-1 viruses were first complexed with lipofectamine (Invitrogen, Carlsbad, CA, USA) as described previously [39] to achieve MoDC transduction efficiencies comparable to described Ad5/3 transduction efficiencies [29] independent of the low expression levels of the Ad5 docking receptor CAR. After 2 h, IMDM complete medium was added to the cells. For eGFP read-out, transduced MoDCs were harvested 48 h later and analyzed for GFP transgene expression in combination with CD86-PE labeled activation marker by flow cytometry as described above (Becton-Dickinson CO, Franklin Lakes, NJ, USA). MoDCs transduced by Ad-MART-1 were used to induce or re-stimulate MART-1-specific CD8^+^ T cells after 24 h of culture.

### 2.6. In Vitro Induction of MART-1-Specific CD8^+^ Effector T Cell 

CD8β^+^ T cells were isolated from PBMC of HLA-A2^+^ healthy donors by positive labeling with anti-CD8β antibody (Beckman Coulter, Brea, CA, USA) and goat-anti-mouse (GaM) magnetic beads (Miltenyi Biotec) using a magnetic cell-sorting device (Miltenyi Biotec) according to the manufacturer’s protocol. In order to induce MART-1-specific cytotoxic T lymphocytes (CTLs), 1 × 10^6^ CD8β^+^ T cells and 1 × 10^6^ irradiated (10 Gy) CD8β^−^ autologous PBMC were co-cultured with 1 × 10^5^ Ad5-, lipofectamine-complexed Ad5- or Ad5/3-transduced DCs for 10 days in Yssel’s medium [40] supplemented with 1% hAB serum (ICN Biochemicals, Zoetermeer, The Netherlands) in the presence of 10 ng/mL IL-6 and 10 ng/mL IL-12 in a 24-well tissue-culture plate. For each donor, six equivalent cultures were initiated per condition. On Day 1 after the start of the experiment, 10 ng/mL IL-10 (R&D Systems, Minneapolis, MN, USA) was added to the cells. From Day 10, CD8^+^ T cell cultures were stimulated every week for 2–3 weeks with 1 × 10^5^ freshly transduced autologous MoDCs in the presence of 5 ng/mL IL-7 (Strathmann Biotec, Hamburg, Germany). On the day of each re-stimulation, a sample of each culture was used to determine the frequencies of MART-1_26–35L_ Tm-binding CD8^+^ T cells in the bulk cultures. Two days after each re-stimulation, 10 IU/mL IL-2 (Strathmann Biotec) was added. MART-1_26–35L_ Tm^+^ CD8^+^ T cell were then isolated by magnetic Tm^+^ flow sorting using PE-labeled Tm and anti-PE magnetic beads (Miltenyi Biotec) in order to expand a specificity-enriched bulk population. To this end, sorted CD8^+^ T cell were stimulated each week with irradiated (10 Gy) feeder-mixes composed of allogeneic PBMCs from two different donors (2:1 ratio with CD8^+^ T cells) and JY cells (1:5 ratio with CD8^+^ T cells) in Yssel’s medium supplemented with 100 ng/mL phytohemagglutinin (PHA, from Murex Biotech, Dartford, UK) and 10 IU/mL IL-7. CD8^+^ T cell bulks were subsequently tested in different functional assays. Using the same procedure, MART-1 CD8^+^ T cell bulks were derived by (re-)stimulating with MART-1_26–35L_ peptide-loaded MoDC, as previously described [39].

### 2.7. Preparation, Transduction and Culture of Sentinel Lymph Node Single-Cell Suspensions

Single-cell suspensions from human SLNs were obtained from melanoma patients after their informed consent without interference in standard diagnostic procedures as described previously [41]. This protocol was approved by the Institute’s Medical ethical committee (IRB) and in accordance with the Declaration of Helsinki. In brief, SLN were bisected in a sterile environment and viable cells were scraped from the SLN cutting surface. SLN cells were then collected in 15 mL sterile ice-cold dissociation medium comprising IMDM medium with 25 mM HEPES buffer (BioWhittaker, Verviers, Belgium) supplemented with 5% FCS, 100 IU/mL sodium-penicillin, 100 μg/mL streptomycin, 2 mM l-glutamine, 0.1% DNAse, and 0.14% Collagenase A (Roche Nederland BV, Woerden, The Netherlands). Scraped cells were transferred to sterile glass flasks containing a stirring bean in a total volume of 30 mL dissociation medium and were placed on a magnet for 45 min at 37 °C in a water bath. Subsequently, the SLN cells were filtered over a 100 µM sterile cell strainer (BD Biosciences) into 50 mL tubes. Flasks were rinsed twice with IMDM complete medium. After centrifugation (1560 rpm for 5 min) single cell suspensions were counted, and further processed. The SLN cells were first tested for HLA-A2 expression by flow cytometry. SLN cells derived from HLA-A2^+^ melanoma patients were seeded at a density of 1 × 10^6^ cells/well of a 24-well plate in 500 μL serum-free medium. SLN cells were then transduced with Ad5/3-MART-1 or Ad5-MART-1 at a MOI of 1000 viral particles based on calculated absolute SLN-DC numbers as described previously [29]. After 2 h of transduction at 37 °C, 2 mL of Yssel’s medium was added to the cells supplemented with 10 ng/mL of IL-7 and cells were cultured for 10 days. The frequencies of MART-1_26–35L_ Tm-binding CD8^+^ T cells were subsequently measured by flow cytometry. For re-stimulation at Day 10, 0.1 × 10^6^ HLA-A2^+^ JY cells per bulk culture were loaded with 10 ng/mL MART-1_26–35L_ peptide in the presence of 3 μg/mL β2-microglobulin (Sigma-Aldrich) in serum free medium and after loading were washed and added to the cultures. Two days after re-stimulation, 10 ng/mL IL-2 and IL-15 were added to the cells. 

### 2.8. Intracellular Interferon γ (IFN-γ) Detection

To evaluate the ability of the generated CD8^+^ T cell to produce IFN-γ upon recognition of a particular target cell, intracellular IFN-γ staining was performed. The melanoma tumor cell lines Mel-AKR, Mel-JKO and the glioblastoma cell line U251 were used as target cells, as well as JY cells loaded with either the relevant MART-1_26–35L_ peptide or an irrelevant (HIV) peptide. Resting CD8^+^ T cells that had not been stimulated for at least one week were co-cultured with target cells at an effector:target cell (E:T) ratio of 2:1 in 96-well round-bottom plates in the presence of GolgiPlug (BD Biosciences). After 4–5 h of stimulation, cells were washed and labeled with PE-conjugated MART-1 tetramers and APC-conjugated anti-CD8 mAb. The BD Cytofix/Cytoperm (BD Biosciences) kit was used according to the manufacturer’s protocol for intracellular staining with FITC-labeled anti-IFNγ Ab (BD Biosciences) and analyzed on a FACSCalibur (BD Biosciences).

### 2.9. Functional Avidity Analysis

To determine the functional avidity of the induced MART-1 specific CD8^+^ T cells generated by Ad5/3-MART-1, intracellular IFN-γ staining was performed as described above. Ad5/3-MART-1 primed and tetramer-MACS-isolated MART-1 specific CD8^+^ T cells were compared with control MART-1_26–35L_ peptides primed and MACS-isolated MART-1 specific CD8^+^ T cells, due to frequencies of MART-1 specific CD8^+^ T cells induced with the Ad5-MART-1 virus that were too low to perform these functional assays. HLA-A2^+^ JY cells were used as stimulator cells after being loaded with serial 1000-fold dilutions (10 µM, 10 nM, 10 pM, 10 fM, and no peptide) of the MART-1_26–35L_ peptide and were co-cultured with CD8^+^ T effector cells at effector:stimulator (E:S) ratios of 1:1. IFN-γ production of stimulated CD8^+^ T cells was evaluated by flow cytometry after staining with PE-conjugated MART-1 tetramer, APC-conjugated CD8 and intracellular staining of FITC-labeled IFN-γ. For visualization, the percentages of IFN-γ producing CD8^+^ T cells at the highest peptide concentration of 10 µM were set at 100% for both conditions.

### 2.10. Cytotoxicity Assay

The cytolytic activity of MART-1-specific CD8^+^ T cells was evaluated by a flow cytometry-based cytotoxicity assay. For this purpose, MACS isolated effector CD8^+^ T cells were labeled with 200 nM CFSE (Molecular Probes, Eugene, OR, USA) for 15 min at 37 °C, washed twice, and co-cultured with target cells at 10:1 and 3:1 E:T ratios in a 48-well plate. The melanoma tumor cell lines Mel-AKR (HLA-A2^+^ MART-1^+^), and Mel-JKO (HLA-A2^−^ MART-1^+^) were used as target cells. After 4 to 5 h at 37 °C, target cells were harvested and stained with 7-amino actinomycin D (7-AAD) (Sigma Aldrich, Saint Louis, MO, USA) for 10 min at 4 °C to distinguish necrotic cells. Acquisition was performed on a FACSCalibur and specific lysis was calculated as described previously [39].

### 2.11. Statistical Analysis

Data were analyzed by two-tailed, paired, or unpaired Student *t* test, or one-way or two-way ANOVA with Tukey post-hoc analysis as indicated in the text using GraphPad Prism 6.0 software (GraphPad Software, La Jolla, CA, USA). Differences were considered significant when *p* < 0.05. 

## 3. Results

### 3.1. Superior Efficiency of MART-1_26–35_ Specific CD8^+^ T Cell Priming by Ad5/3-MART-1 Transduced MoDCs as Compared to Ad5-MART-1 Transduced MoDCs

**Figure 1:**
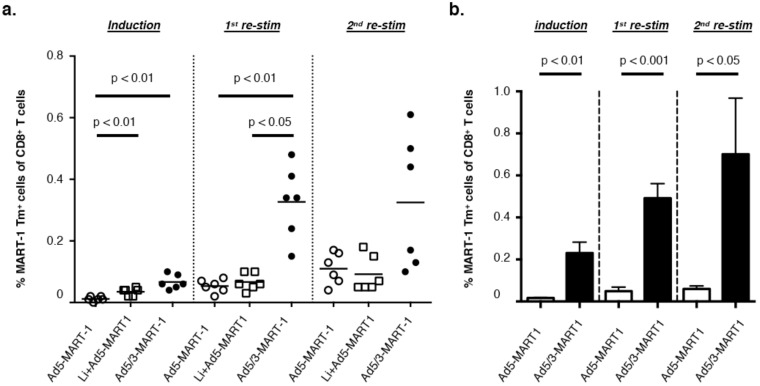
Ad5/3-MART-1 transduced monocyte-derived dendritic cells (MoDCs) more efficiently prime and expand MART-1 Tm^+^ CD8^+^ T cells than Ad5-MART-1 transduced MoDC. (**a**) MART-1 induction and re-stimulation results of a representative donor using mature MoDC transduced with Ad5-MART-1, Lipofectamine (Li)-complexed Ad5-MART-1 or Ad5/3-MART-1. Six bulk cultures were started for each condition. Frequencies of Tm^+^ (MART-1_26–35L_) CD8^+^ T cells were analyzed on Day 10 (induction), Day 18 (1st re-stim) and Day 25 (2nd re-stim). One-way ANOVA with Tukey multiple comparison analyses was performed to determine statistical significance. (**b**) Combined data for Ad5-MART-1 versus Ad5/3-MART-1 priming from n = 4 experiments with six bulk cultures per condition per experiment. Unpaired Student *t*-tests were performed between Ad5-MART-1 and Ad5/3-MART-1 conditions for each time point.

We investigated the capability of autologous mature MoDCs transduced by MART-1 encoding Ad5/3 to prime TAA-specific, HLA-A2restricted CTL. Ad5/3 and Ad5 vectors encoding the full-length MART-1 antigen were used for transduction of mature MoDCs (MOI 1000 vp). To obtain comparable transduction efficiencies between Ad5-MART-1 and Ad5/3-MART-1, an additional control transduction was taken along in two experiments, complexing Ad5-MART-1 to lipofectamine prior to MoDC transduction [39]. As a read-out, we used a fluorescently labeled tetramer recognizing the high-affinity altered-peptide ligand for the immune-dominant MART-1 epitope MART-1_26–35_; the MART-1_26–35L_ [42]. MART-1_26–35L_-specific T cells could be detected at higher frequencies in cultures stimulated with MoDCs transduced by Ad5/3-MART-1 compared to Ad5-MART-1 or lipofectamine-complexed Ad5-MART-1 after the induction phase and after the first re-stimulation (Figure 1a). After two rounds of in vitro re-stimulation with Ad-transduced autologous MoDCs, the frequencies of MART-1-specific CD8^+^ T cells stimulated by Ad5/3-MART-1 transduced MoDCs were slightly further increased as shown for a representative donor (Figure 1a) and combined data for four donors (Figure 1b).

### 3.2. T Cells Primed with Ad5/3-MART-1 Transduced MoDCs Recognize Endogenously Processed MART-1

**Figure 2:**
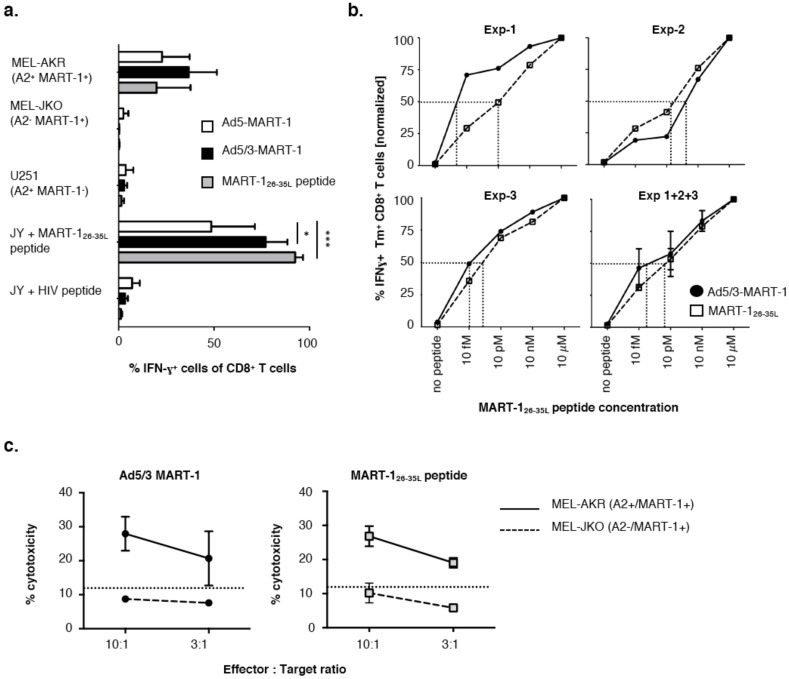
Ad5/3-MART-1 cytotoxic T lymphocyte specificity, avidity and cytotoxicity. (**a**) MART-1 specific CD8^+^ T cells primed by Ad5-MART-1, Ad5/3-MART-1-transduced MoDCs or MART-1_26–35L_ peptide-loaded MoDCs produced IFN-γ upon recognition of endogenously processed MART-1 protein in the context of HLA-A2 (MEL-AKR) or specific peptide-loaded HLA-A2^+^ JY cells, but were unable to recognize irrelevant peptides or HLA-A2^−^ MART-1^+^ (MEL-JKO) or HLA-A2^+^ MART-1^−^ (U251) targets. Combined results from three priming experiments with independent donors are shown. A two-way ANOVA with Tukey multiple comparison analysis was performed, showing statistically significant differences in the recognition of MART-1_26–35L_-loaded JY cells by Ad5/3-MART-1 induced CD8^+^ T cells (*p* < 0.05) or MART-1_26–35L_ peptide-induced CD8^+^ T cells (*p* < 0.001) compared to Ad5-MART-1-induced CD8^+^ T cells. (**b**) HLA-A2^+^ JY cells were loaded with titrated MART-1_26–35L_ peptide as indicated and cultured with Ad5/3-MART-1 primed or MART-1_26–35L_ peptide primed CD8^+^ T cells for 4–5 h in the presence of golgiplug. Avidity was assessed by means of intracellular IFN-γ staining. IFN-γ release upon recognition of JY cells loaded with 10 µM peptide was set at 100% for both Ad5/3-MART-1 and MART-1_26–35L_ peptide-induced CD8^+^ T cells. Graphs are shown for three separate experiments with MART-1_26–35L_ specific CD8^+^ T cells generated from independent donors, as well as a graph showing the combined values of experiment 1 + 2 + 3 (bottom right) (means ± SEM) Half maximum release levels are indicated by the dotted lines. (**c**) Expanded and isolated MART-1 specific CD8^+^ T cells primed by Ad5/3-MART-1 transduced MoDCs (left) or MART-1_26–35L_ peptide-loaded MoDCs (right) were able to kill MART-1 expressing tumor cells in a HLA-A2 restricted manner. Averaged data from two separate experiments with CD8^+^ T cells derived from two different HLA-A2^+^ donors are shown (mean ± SEM).

To evaluate the functionality of the MART-1-specific CD8^+^ T cells, we assessed their ability to recognize endogenously processed MART-1 epitopes. For this purpose, the MART-1-specific CD8^+^ T cells that were induced by stimulation with Ad5-MART-1 or Ad5/3-MART-1 transduced or obtained after priming and restimulation with control MART-1_26–35L_ peptide loaded MoDCs were isolated by magnetic MART-1_26–35L_ Tm PE sorting and were expanded by several rounds of re-stimulation with feeder-mix. Of note, insufficient numbers of specific CD8^+^ T cells were obtained through this method for Ad5-MART-1-transduced MoDCs to perform all functional analyses. CD8^+^ T cells were co-cultured for 4 h with different target cells: a MART-1^+^ HLA-A2^+^ cell line (Mel-AKR), a MART-1^+^ HLA-A2^−^ cell line (Mel-JKO), a MART-1^−^ HLA-A2^+^ cell line (U251), and HLA-A2^+^ JY cells loaded with MART-1_26–35L_ peptide or an irrelevant HIV peptide. The CD8^+^ T cells were assessed for IFN-γ production in order to determine the recognition of the endogenously processed MART-1_26–35_ epitope (Figure 2a). MART-1-specific T-cells induced by Ad-5/3-MART-1 and Ad5-MART-1 transduced MoDCs or by MART-1_26–35L_ peptide-loaded MoDCs were all capable of recognizing the HLA-A2^+^ JY target cells loaded with the specific MART-1_26–35L_ peptide with high specificity, but not the HIV peptide-loaded JY targets. In addition, the CD8^+^ T cells also exclusively recognized the HLA-A2^+^MART-1^+^ Mel-AKR cell line, demonstrating their ability to specifically recognize the endogenously processed MART-1 epitope restricted by HLA-A2. MART-1-specific CD8^+^ T cells stimulated by Ad5/3-MART-1-transduced MoDCs produced higher levels of IFN-γ in response to Mel-AKR than those stimulated by Ad5-MART-1-transduced MoDCs or MART-1_26–35L_-pulsed MoDCs; however, this difference did not reach statistical significance (Figure 2a). Note that IFN-γ production in response to MART-1 peptide loaded targets was significantly elevated in Ad5/3 primed CD8^+^ T cells over that of their Ad5 primed counterparts (Figure 2a).

### 3.3. Functional Avidity of MART-1_26–35L_-Specific CD8^+^ T Cells Primed by Ad5/3-MART-1 Targeted MoDCs

Expanded MART-1-specific CD8^+^ T cells that were induced through stimulation with either Ad5/3 transduced or peptide-loaded MoDCs were evaluated for their functional avidity. Primed CD8^+^ T cells were co-cultured with HLA-A2^+^ JY cells loaded with different concentrations of MART-1_26–35L_ peptide. Intracellular IFN-γ production by MART-1_26–35L_ tetramer-isolated CD8^+^ T cells was used as a read-out of this assay. In two out of three donors tested, MART-1-specific CD8^+^ T cells induced by Ad5/3-MART-1-transduced DCs demonstrated a higher functional avidity than CD8^+^ T cells induced by MART-1_26–35L_ peptide pulsed DCs (Figure 2b, Exp-1 and Exp-3). In the other donor tested, the CD8^+^ T cells primed by MART1_26–35L_ peptide-loaded MoDCs displayed a slightly higher functional avidity (Figure 2b, Exp-2). Based on three separate experiments with three independent donors, comparable production levels of IFN-γ reflected the equivalent recognition of the MART-1_26–35L_ epitope by CD8^+^ T cells primed with MoDCs transduced by Ad5/3-MART-1, encoding full-length MART-1, or with MART-1_26–35L_ peptide-pulsed MoDCs. Figure 2b also shows the combined data of three separate priming experiments, displaying relative IFN-γ percentages, with percentages for the highest tested concentration (10 µM) set at 100% per condition. The actual percentages of IFN-γ producing Tm^+^ CD8^+^ T cells for the 10 µM concentration were 91.2 ± 7.7% and 80.2 ± 13.2% for MART-1_26–35L_ peptide-generated and Ad5/3-MART-1-generated CTL, respectively. The dotted lines in Figure 2b indicate the EC50 value (half-maximum) of IFN-γ production, which was slightly lower, though not statistically significant, for Ad5/3-MART-1 primed CD8^+^ T cells than for MART-1_26–35L_ peptide-primed CD8^+^ T cells when looking at the combined data of three experiments.

### 3.4. Melanoma Cytolysis by T Cells Primed with Ad5/3-MART-1 Transduced MoDCs

In agreement with the IFN-γ release experiments, MART-1-specific CD8^+^ T cells generated through Ad5/3-MART-1 transduced or MART-1_26–35L_ peptide-pulsed MoDCs showed specific cytolytic activity against the HLA-A2^+^ MART-1^+^ MEL-AKR tumor cell line, but not the HLA-A2^−^ MART-1^+^ MEL-JKO cell line (Figure 2c). Data shown are average cytotoxicity percentages for two independent CTL donors. A third experiment also showed specific cytotoxicity, but for both the Ad5/3-MART-1 CTL and MART-1_26–35L_ peptide CTL maximum killing was below 10%.

### 3.5. Superior Expansion of MART-1 Specific CD8^+^ T Cells Residing in Melanoma SLN Upon Ad5/3-MART-1 Infection as Compared to Ad5-MART-1

**Figure 3:**
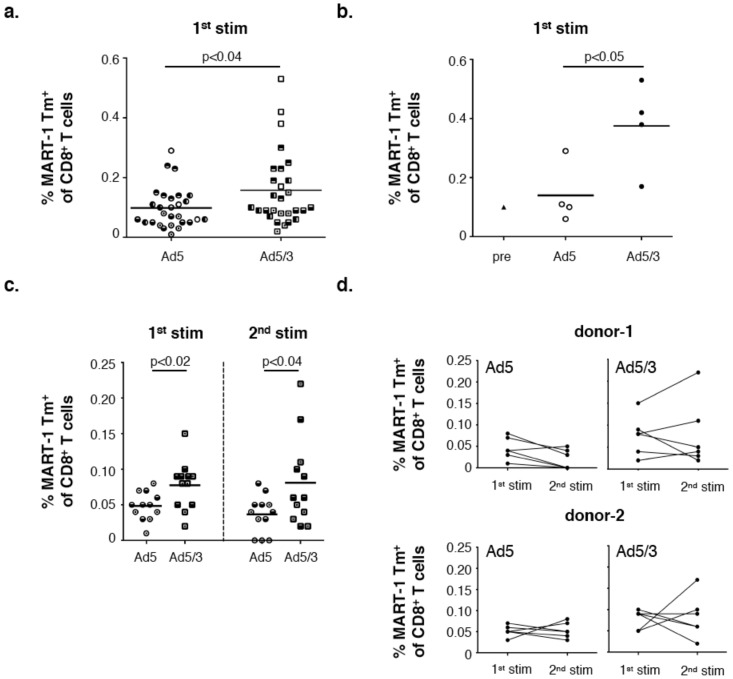
In situ stimulation of MART-1 specific CD8^+^ T cells in melanoma sentinel lymph nodes (SLNs). Stimulation of pre-existing MART-1 specific CD8^+^ T cells present within metastasis negative melanoma sentinel lymph node single-cell suspensions (HLA-A2^+^) through ex vivo infection using either Ad5-MART-1 or Ad5/3-MART-1. (**a**) Combined data showing MART-1_26–35L_ Tm^+^ CD8^+^ T cells of in total 28 bulk cultures upon primary stimulation (n = 5 SLN donors) with bulk cultures from the same donor indicated with the same filling patterns. An unpaired Student t test was performed between the Ad5-MART-1 and Ad5/3-MART-1 conditions; (**b**) Precursor frequency of MART-1_26–35L_ Tm^+^ CD8^+^ T cells in SLN donor-5 compared to frequencies after one round of ex vivo stimulation (d10) with Ad5-MART-1 or Ad5/3-MART-1; (**c**) Depicted are the percentages of MART-1_26–35L_ Tm^+^ cells from CD8^+^ T cells after the primary stimulation with Ad5-MART-1 or Ad5/3-MART-1 (Day 11) and after one round of re-stimulation with irradiated MART-1_26–35L_ loaded HLA-A2^+^ JY cells (Day 18) (n = 2 donors 1+2, 6 bulk cultures per donor per condition). (**d**) Donors depicted in (**c**) separated for Ad5-MART-1 and Ad5/3-MART-1 stimulation showing increased or decreased frequencies of MART-1 Tm^+^ cells of CD8^+^ T cells between the primary (1st stim) and secondary (2nd stim) ex vivo stimulation. In this figure Ad5 = Ad5-MART-1, Ad5/3 = Ad5/3-MART-1.

The ability of Ad5 and Ad5/3 to selectively and efficiently transduce DCs was previously evaluated by our group in ex vivo LN suspensions. It was shown that Ad5/3-eGFP transduction of DCs in melanoma-draining SLN led to significantly enhanced eGFP transgene expression levels compared to Ad5-eGFP-mediated transduction, especially of the more mature skin-derived, migratory DC subsets present in skin-draining SLN [41]. Regarding the melanoma SLN as the primary site for melanoma-specific T cell induction, here we investigated the ability of Ad5/3-MART-1 transduced SLN-APCs to expand melanoma-reactive T cells residing within SLN single-cell suspensions as compared to Ad5-MART-1. Our data showed increased frequencies of MART-1-specific CD8^+^ T cell (recognizing the MART-1_26–35_ epitope) in SLN, upon ex vivo stimulation through Ad5/3-MART-1 transduction as compared to Ad5-MART-1 (Figure 3). Figure 3a shows the frequencies of MART-1 Tm^+^ CD8^+^ T cells from n = 5 SLN after the primary stimulation. Figure 3b shows for one donor the precursor frequency of MART-1_26–35L_ Tm^+^ CD8^+^ T cells within the single cell suspension and the corresponding frequencies after the primary ex vivo stimulation using Ad5-MART-1 or Ad5/3-MART-1. Graphs in Figure 3c,d show the primary stimulation and further re-stimulation data from two donors, with six bulk cultures per donor. Due to restrictively low rates of MART-1 Tm^+^ CD8^+^ T cells in these short-term cultures, further functional assays could not be performed.

## 4. Discussion

With the recent, accumulating successes of checkpoint inhibitors in treating advanced stage melanoma as well as a growing number of other tumor indications [1,2,43], new challenges have emerged. Firstly, predictive biomarkers need to be identified that can aid in selecting those patients that are most likely to respond to these type of therapies. Secondly, the cancer field is challenged to find combination treatments that will result in increased response rates on immunotherapy, particularly for those patients that have low frequencies of type-1 and effector memory tumor-infiltrating lymphocytes. With this challenge, a renewed interest in vaccine strategies has emerged that can induce or expand antitumor effector T cells in vivo, leading to increased tumor-infiltrating T cell numbers [44]. One of the first steps of getting T cells into the tumor microenvironment is efficient presentation of tumor-antigens by DCs and subsequent priming of naïve T cells [45]. We investigated whether the use of a MART-1 expressing chimeric Ad5/3, targeting CD80 and CD86 on DCs, could result in efficient priming and activation of melanoma specific CD8^+^ T effector cells both in vitro and in a human ex vivo SLN model. Indeed, DCs transduced by this CD80/CD86-targeted Ad vector significantly enhanced both the priming efficiency of MART-1 specific CD8^+^ T effector cells and expansion of TAA-reactive CD8^+^ T cells in SLN, as compared to DCs transduced by a MART-1 expressing serotype-5 adenoviral vector (Ad5-MART-1). Moreover, results showed that the generated MART-1-recognizing CD8^+^ T cells were fully functional and capable of recognizing and killing melanoma tumor cells, as effectively as MART-1 specific CD8^+^ T cells primed by MART-1_26–35L_ peptide-pulsed DCs. CD8^+^ T cells from all test conditions were able to specifically recognize HLA-A2^+^ targets exogenously loaded with the MART-1_26–35L_ epitope or a HLA-A2^+^ melanoma cell line expressing endogenously processed MART-1 epitopes. Of note, CD8^+^ T cells primed with Ad5/3-MART-1-transduced DCs showed a trend towards higher production levels of the immunostimulatory cytokine IFN-γ in response to HLA-A2^+^ melanoma cells expressing MART-1 as compared to the other in vitro primed CD8^+^ T cells. Moreover, the CD8^+^ T cells primed by Ad5/3-targeted DCs also demonstrated specific cytotoxic activity against melanoma cells expressing endogenously processed MART-1 epitopes in an HLA-A2-restricted fashion. As a general consensus, high avidity CD8^+^ T effector cells mediate better antitumor T cell responses with higher efficacy to eliminate tumor cells [46].

MoDCs pulsed with the immunodominant HLA-A2-restricted epitope MART-1_26–35L_, an altered peptide ligand with high affinity major histocompatibility complex-binding properties, could significantly induce high frequencies of MART-1 specific CTLs [39]. However, the fact that Ad vectors encoding the full-length MART-1 protein were used for DC transduction and all our read-out assays were conducted with a single MART-1 epitope, reveals that highly efficient antigen processing and presentation occurred in the transduced DCs for subsequent CD8^+^ T cell stimulation. The frequencies of MART-1_26–35L_ Tm^+^ CD8^+^ T cells present in the bulk cultures upon Ad5/3-MART-1 stimulation, likely under-represents the actual frequencies of MART-1 protein specific effector CD8^+^ T cells in these cultures. The enhanced cytolysis of Mel-AKR tumor cells by Ad5/3-MART-1 induced CTLs is remarkable and also suggests very efficient recognition of the endogenously processed MART-1_26–35wt_ epitope. In this context, the use of recombinant Ad encoding for the full-length MART-1 sequence has definite benefits over the use of a single peptide epitope, as multiple epitopes could be presented on various HLA alleles, including Th and CTL epitopes, and applied as a vaccine without HLA restrictions. Furthermore, from a clinical perspective, in vivo administration of short peptides like the MART-1_26–35L_ epitope could lead to their direct binding to major histocompatibility (MHC) molecules on the surface of non-professional APCs, thereby potentially inducing tolerance or anergy in the absence of correct co-stimulation [47]. In addition, the limited or absent tertiary structure of short peptides makes them susceptible to exopeptidase-mediated degradation [22,48]. In contrast, adenoviruses are stable gene delivery vehicles in vivo, promoting optimal antigen presentation by DCs in secondary lymphoid organs or at the site of the tumor.

In vivo DC targeting approaches often focus on targeting easily accessible peripheral DCs in skin. However, intradermally delivered Ad viruses may also drain directly to LNs and target DC subsets there [49]. In melanoma, DC populations within the SLN have been reported to express lower levels of activation markers because of a generally less activated phenotype compared to DCs in second-line melanoma-draining LNs [23,30]. In this context, SLN may be characterized by their restrained ability to generate protective anti-tumor T cell immunity. We however previously showed that especially the more matured, skin-migrated DC subsets present within SLN, still express sufficiently high levels of the co-stimulatory molecules CD80 and CD86, to be efficiently targeted by Ad3 knob-containing viruses [29,41]. The results from the current study show that ex vivo transduction of DCs in SLN cell suspensions from melanoma patients by Ad5/3-MART-1 facilitated the expansion of TAA-specific CD8^+^ T cells leading to higher frequencies of MART-1_26–35L_-specific T cells compared to the wild-type Ad5-MART-1. Unfortunately, it was not possible to evaluate these MART-1_26–35L_-specific T cells for their functional capacities, since expansion of T cells in these short-term cultures did not reach numbers compatible with functional assays. T cell receptor (TCR) sequencing studies could explore whether the expanded MART-1 specific T cells within such SLN suspensions represent clonal T cell populations. Moreover, since no functional assays could be performed with the T cells expanded in these cultures, we do not know whether those cells are functionally capable of producing IFN-γ and killing their target cells similarly to the MART-1_26–35L_ specific T cells generated through in vitro priming experiments using MoDC. Their expansion as well as functional capabilities could potentially be improved through the addition of checkpoint inhibitors like anti-CTLA4 and/or anti-PD-1. However, as with all checkpoint inhibitor-containing immunotherapy regimens, the development of any immune adverse events should be closely monitored should such combination therapies be tested in vivo.

Although chimeric fiber-modified Ad5/3 appears to be an appealing vaccine vector, various challenges relating to its clinical translation need to be addressed in order to obtain clinical proof-of-concept [32]. The principal concern is related to the fact that Ad5- or Ad2-based vaccines can often only be applied once as an immediately induced or enhanced Ad-specific immune response will neutralize a booster vaccine [25,32]. This does not only involve pre-existing Ad-neutralizing Abs, but also pre-existing Ad-specific CTL responses. Indeed Molinier-Frenkel et al. [50] have shown that despite deleting adenoviral regions (E1, E1 + E2, or E1 + E4), anti-viral CTL responses are still activated, already upon the first injection in individuals with pre-existing immunity. Since in our system, the Ad5/3-MART-1 is targeted to CD80/CD86-expressing DCs, this could result in the stimulation of Ad-specific T cell responses through the presentation of viral antigens, and subsequent killing of the Ad-transduced DCs and quenching of the anti-MART-1 response over time. We did not assess the magnitude of the Ad-directed CD8^+^ T cell response in the SLN cultures. We anticipate that the proposed local (intradermal) administration of Ad-vectors targeting DC subsets in the tumor/skin-draining lymph nodes will be less hampered by anti-Ad cellular immune responses as it was shown that this route of administration in human healthy individuals, using Ad5 vectors, resulted only in mild to moderate local cellular immune responses, even in immunized individuals [51]. Moreover, to overcome this hurdle of pre-existing immunity, scientists have shifted their focus from homologous to heterologous prime-boost strategies. In this case, Ads of different serotypes are used for the priming or the boosting step. It has been found that heterologous prime-boost strategies led to more robust Th1 cellular responses in mice and non-human primates [33,34]. Kahl et al. [35] demonstrated that priming with Ad5 was most efficient when a rare Ad serotype with low seroprevalence in the general population, such as Ad28, was used as a booster vaccine. In this context, many chimeric Ad5 vectors have been designed by replacing the Ad5 fiber domain or just the fiber knob with a fiber of less common human serotypes [52,53,54] or of non-human serotypes [55,56,57]. The Ad3 serotype is one of the most widely studied for altering Ad5 tropism with relatively low seroprevalence [58] and use of CD80/CD86 as primary docking receptors [28,32]. Our data suggest chimeric fiber-modified Ad5/3, with high efficiency in DC transduction, to be a good candidate for prime-boost vaccine strategies. For this purpose, a panel of different, but equally efficient DC-targeting Ad vectors, such as Ad3 [29,34,35], Ad35 [59], and CD40-Ad5 [39,60], should be tested in different prime-boost combination vaccination schemes in order to promote the generation of an even more robust antigen-specific T cell response [61,62,63,64,65] without collateral and restrictive anti-Ad-immunity. As it has been reported that Ad5 knob-swap variants could still be neutralized by Ad5-specific antibodies that mostly bind the Ad5 hexon protein [39,66,67], hexon-chimera vectors [68,69,70] or the different wild type (wt) serotype vectors (e.g., Ad3, Ad11, Ad35) might be more efficacious for clinical use [71]. Combination of Ad-based vaccines with protein, DNA or RNA vaccines, or bacterial or other viral vectors could also be used in prime-boost strategies in order to eliminate negative effects of anti-Ad immunity [72]. In addition to targeting DCs via CD80/CD86, Ad3-based oncolytic adenoviruses are currently being developed for clinical use to directly target the tumor microenvironment, through binding of Ad3 to tumor-expressed desmoglein-2, a receptor used by various type B adenoviruses and expressed on several cancer types [29,41,73,74,75]. These oncolytic adenoviruses are also being armed to include DC-activating components such as CD40-ligand [76]. In conclusion, Ad3-based viruses, be it replication-incompetent or competent, seem very useful in promoting antitumor effector T cell responses either via oncolysis or targeting to DCs and could be used combined with immune checkpoint inhibitors like anti-CTLA-4 or anti-PD(L)-1 to overcome the suppression of tumor-specific T cells during the priming phase (anti-CTLA-4) or the effector phase in the tumor microenvironment (anti-PD-(L)1).

## 5. Conclusions

Collectively, our data showing the improved expansion of MART-1 specific CD8^+^ effector T cells both from naïve blood and from melanoma-draining SLN support the use of Ad3-based viruses as an in vivo DC-targeting vaccine vehicle in patients with melanoma.

## References

[1] Hodi F.S., O’Day S.J., McDermott D.F., Weber R.W., Sosman J.A., Haanen J.B., Gonzalez R., Robert C., Schadendorf D., Hassel J.C. (2010). Improved survival with ipilimumab in patients with metastatic melanoma. N. Engl. J. Med..

[2] Wolchok J.D., Kluger H., Callahan M.K., Postow M.A., Rizvi N.A., Lesokhin A.M., Segal N.H., Ariyan C.E., Gordon R.-A., Reed K. (2013). Nivolumab plus ipilimumab in advanced melanoma. N. Engl. J. Med..

[3] Topalian S.L., Sznol M., McDermott D.F., Kluger H.M., Carvajal R.D., Sharfman W.H., Brahmer J.R., Lawrence D.P., Atkins M.B., Powderly J.D. (2014). Survival, durable tumor remission, and long-term safety in patients with advanced melanoma receiving nivolumab. J. Clin. Oncol..

[4] Ascierto P.A., Capone M., Urba W.J., Bifulco C.B., Botti G., Lugli A., Marincola F.M., Ciliberto G., Galon J., Fox B.A. (2013). The additional facet of immunoscore: Immunoprofiling as a possible predictive tool for cancer treatment. J. Transl. Med..

[5] Chen D.S., Mellman I. (2013). Oncology Meets Immunology: The Cancer-Immunity Cycle. Immunity.

[6] Gubin M.M., Zhang X., Schuster H., Caron E., Ward J.P., Noguchi T., Ivanova Y., Hundal J., Arthur C.D., Krebber W.-J. (2014). Checkpoint blockade cancer immunotherapy targets tumour-specific mutant antigens. Nature.

[7] Schumacher T.N., Schreiber R.D. (2015). Neoantigens in cancer immunotherapy. Science.

[8] Butterfield L.H., Comin-Anduix B., Vujanovic L., Lee Y., Dissette V.B., Yang J.-Q., Vu H.T., Seja E., Oseguera D.K., Potter D.M. (2008). Adenovirus MART-1-engineered autologous dendritic cell vaccine for metastatic melanoma. J. Immunother..

[9] Banchereau J., Palucka A.K. (2005). Dendritic cells as therapeutic vaccines against cancer. Nat. Rev. Immunol..

[10] Redman B.G., Chang A.E., Whitfield J., Esper P., Jiang G., Braun T., Roessler B., Mulé J.J. (2008). Phase Ib trial assessing autologous, tumor-pulsed dendritic cells as a vaccine administered with or without IL-2 in patients with metastatic melanoma. J. Immunother..

[11] Verdijk P., Aarntzen E.H.J.G., Lesterhuis W.J., Boullart A.C.I., Kok E., van Rossum M.M., Strijk S., Eijckeler F., Bonenkamp J.J., Jacobs J.F.M. (2009). Limited amounts of dendritic cells migrate into the T-cell area of lymph nodes but have high immune activating potential in melanoma patients. Clin. Cancer Res..

[12] Fay J.W., Palucka A.K., Paczesny S., Dhodapkar M., Johnston D.A., Burkeholder S., Ueno H., Banchereau J. (2006). Long-term outcomes in patients with metastatic melanoma vaccinated with melanoma peptide-pulsed CD34(+) progenitor-derived dendritic cells. Cancer Immunol. Immunother..

[13] Tacken P.J., de Vries I.J.M., Torensma R., Figdor C.G. (2007). Dendritic-cell immunotherapy: From ex vivo loading to in vivo targeting. Nat. Publ. Group.

[14] Steinman R.M. (2008). Dendritic cells in vivo: A key target for a new vaccine science. Immunity.

[15] Mahnke K., Qian Y., Fondel S., Brueck J., Becker C., Enk A.H. (2005). Targeting of antigens to activated dendritic cells in vivo cures metastatic melanoma in mice. Cancer Res..

[16] Breckpot K., Aerts J.L., Thielemans K. (2007). Lentiviral vectors for cancer immunotherapy: Transforming infectious particles into therapeutics. Gene Ther..

[17] Altin J.G., Parish C.R. (2006). Liposomal vaccines—Targeting the delivery of antigen. Methods.

[18] Khare R., Chen C.Y., Weaver E.A., Barry M.A. (2011). Advances and future challenges in adenoviral vector pharmacology and targeting. Curr. Gene Ther..

[19] Lotem M., Zhao Y., Riley J., Hwu P., Morgan R.A., Rosenberg S.A., Parkhurst M.R. (2006). Presentation of tumor antigens by dendritic cells genetically modified with viral and nonviral vectors. J. Immunother..

[20] Tuettenberg A., Jonuleit H., Tüting T., Brück J., Knop J., Enk A.H. (2003). Priming of T cells with Ad-transduced DC followed by expansion with peptide-pulsed DC significantly enhances the induction of tumor-specific CD8+ T cells: Implications for an efficient vaccination strategy. Gene Ther..

[21] Zhai Y., Yang J.C., Kawakami Y., Spiess P., Wadsworth S.C., Cardoza L.M., Couture L.A., Smith A.E., Rosenberg S.A. (1996). Antigen-specific tumor vaccines. Development and characterization of recombinant adenoviruses encoding MART1 or gp100 for cancer therapy. J. Immunol..

[22] Rea D., Havenga M.J., van Den Assem M., Sutmuller R.P., Lemckert A., Hoeben R.C., Bout A., Melief C.J., Offringa R. (2001). Highly efficient transduction of human monocyte-derived dendritic cells with subgroup B fiber-modified adenovirus vectors enhances transgene-encoded antigen presentation to cytotoxic T cells. J. Immunol..

[23] Noureddini S.C., Curiel D.T. (2005). Genetic targeting strategies for adenovirus. Mol. Pharm..

[24] Smith J.S., Tian J., Lozier J.N., Byrnes A.P. (2004). Severe pulmonary pathology after intravenous administration of vectors in cirrhotic rats. Mol. Ther..

[25] Aichele P., Brduscha-Riem K., Zinkernagel R.M., Hengartner H., Pircher H. (1995). T cell priming versus T cell tolerance induced by synthetic peptides. J. Exp. Med..

[26] Raki M., Sarkioja M., Escutenaire S., Kangasniemi L., Haavisto E., Kanerva A., Cerullo V., Joensuu T., Oksanen M., Pesonen S. (2011). Switching the fiber knob of oncolytic adenoviruses to avoid neutralizing antibodies in human cancer patients. J. Gene Med..

[27] Fontana L., Nuzzo M., Urbanelli L., Monaci P. (2003). General strategy for broadening adenovirus tropism. J. Virol..

[28] Short J.J., Pereboev A.V., Kawakami Y., Vasu C., Holterman M.J., Curiel D.T. (2004). Adenovirus serotype 3 utilizes CD80 (B7.1) and CD86 (B7.2) as cellular attachment receptors. Virology.

[29] Van de Ven R., Lindenberg J.J., Oosterhoff D., van den Tol M.P., Rosalia R.A., Murakami M., Everts M., Scheffer G.L., Scheper R.J., de Gruijl T.D. (2009). Selective transduction of mature DC in human skin and lymph nodes by CD80/CD86-targeted fiber-modified adenovirus-5/3. J. Immunother..

[30] Cochran A.J., Morton D.L., Stern S., Lana A.M., Essner R., Wen D.R. (2001). Sentinel lymph nodes show profound downregulation of antigen-presenting cells of the paracortex: Implications for tumor biology and treatment. Mod. Pathol..

[31] Cochran A.J., Huang R.-R., Lee J., Itakura E., Leong S.P.L., Essner R. (2006). Tumour-induced immune modulation of sentinel lymph nodes. Nat. Rev. Immunol..

[32] De Gruijl T.D., van de Ven R. (2012). Chapter six—Adenovirus-based immunotherapy of cancer: Promises to keep. Adv. Cancer Res..

[33] Radosević K., Rodriguez A., Lemckert A.A.C., van der Meer M., Gillissen G., Warnar C., von Eyben R., Pau M.G., Goudsmit J. (2010). The Th1 immune response to Plasmodium falciparum circumsporozoite protein is boosted by adenovirus vectors 35 and 26 with a homologous insert. Clin. Vaccine Immunol..

[34] Rodriguez A., Mintardjo R., Tax D., Gillissen G., Custers J., Pau M.G., Klap J., Santra S., Balachandran H., Letvin N.L. (2009). Evaluation of a prime-boost vaccine schedule with distinct adenovirus vectors against malaria in rhesus monkeys. Vaccine.

[35] Kahl C.A., Bonnell J., Hiriyanna S., Fultz M., Nyberg-Hoffman C., Chen P., King C.R., Gall J.G.D. (2010). Potent immune responses and in vitro pro-inflammatory cytokine suppression by a novel adenovirus vaccine vector based on rare human serotype 28. Vaccine.

[36] Molenkamp B.G., Sluijter B.J.R., van Leeuwen P.A.M., Santegoets S.J.A.M., Meijer S., Wijnands P.G.J.T.B., Haanen J.B.A.G., van den Eertwegh A.J.M., Scheper R.J., de Gruijl T.D. (2008). Local administration of PF-3512676 CpG-B instigates tumor-specific CD8^+^ T-cell reactivity in melanoma patients. Clin. Cancer Res..

[37] Heemskerk M.H., Hooijberg E., Ruizendaal J.J., van der Weide M.M., Kueter E., Bakker A.Q., Schumacher T.N., Spits H. (1999). Enrichment of an antigen-specific T cell response by retrovirally transduced human dendritic cells. Cell. Immunol..

[38] Schreurs M.W.J., Scholten K.B.J., Kueter E.W.M., Ruizendaal J.J., Meijer C.J.L.M., Hooijberg E. (2003). In vitro generation and life span extension of human papillomavirus type 16-specific, healthy donor-derived CTL clones. J. Immunol..

[39] Hangalapura B.N., Oosterhoff D., Aggarwal S., Wijnands P.G.J.T.B., van de Ven R., Santegoets S.J.A.M., van den Tol M.P., Hooijberg E., Pereboev A., van den Eertwegh A.J.M. (2010). Selective transduction of dendritic cells in human lymph nodes and superior induction of high-avidity melanoma-reactive cytotoxic T cells by a CD40-targeted adenovirus. J. Immunother..

[40] Yssel H., De Vries J.E., Koken M., Van Blitterswijk W., Spits H. (1984). Serum-free medium for generation and propagation of functional human cytotoxic and helper T cell clones. J. Immunol. Methods.

[41] Van de Ven R., van den Hout M.F.C.M., Lindenberg J.J., Sluijter B.J.R., van Leeuwen P.A.M., Lougheed S.M., Meijer S., van den Tol M.P., Scheper R.J., de Gruijl T.D. (2011). Characterization of four conventional dendritic cell subsets in human skin-draining lymph nodes in relation to T-cell activation. Blood.

[42] Santegoets S.J.A.M., Bontkes H.J., Stam A.G.M., Bhoelan F., Ruizendaal J.J., van den Eertwegh A.J.M., Hooijberg E., Scheper R.J., de Gruijl T.D. (2008). Inducing antitumor T cell immunity: Comparative functional analysis of interstitial versus Langerhans dendritic cells in a human cell line model. J. Immunol..

[43] Luke J.J., Hodi F.S. (2013). Ipilimumab, vemurafenib, dabrafenib, and trametinib: Synergistic competitors in the clinical management of BRAF mutant malignant melanoma. Oncologist.

[44] Van der Burg S.H. (2018). Correlates of immune and clinical activity of novel cancer vaccines. Semin. Immunol..

[45] Church S.E., Galon J. (2017). Regulation of CTL Infiltration within the Tumor Microenvironment. Adv. Exp. Med. Biol..

[46] Dutoit V., Rubio-Godoy V., Dietrich P.Y., Quiqueres A.L., Schnuriger V., Rimoldi D., Liénard D., Speiser D., Guillaume P., Batard P. (2001). Heterogeneous T-cell response to MAGE-A10(254-262): High avidity-specific cytolytic T lymphocytes show superior antitumor activity. Cancer Res..

[47] Toes R.E., Offringa R., Blom R.J., Melief C.J., Kast W.M. (1996). Peptide vaccination can lead to enhanced tumor growth through specific T-cell tolerance induction. Proc. Natl. Acad. Sci. USA.

[48] Slingluff C.L. (2011). The present and future of peptide vaccines for cancer: Single or multiple, long or short, alone or in combination?. Cancer J..

[49] Labow D., Lee S., Ginsberg R.J., Crystal R.G., Korst R.J. (2000). Adenovirus vector-mediated gene transfer to regional lymph nodes. Hum. Gene Ther..

[50] Molinier-Frenkel V., Gahery-Segard M., Le Boulaire C., Ribault S., Boulanger P., Tursz T., Guillet J.G., Farace F. (2000). Immune response to recombinant adenovirus in humans: Capsid components from viral input are targets for vector-specific cytotoxic T lymphocytes. J. Virol..

[51] Harvey B.G., Worgall S., Ely S., Leopold P.L., Crystal R.G. (1999). Cellular immune responses of healthy individuals to intradermal administration of an E1-E3-adenovirus gene transfer vector. Hum. Gene Ther..

[52] Barouch D.H., Kik S.V., Weverling G.J., Dilan R., King S.L., Maxfield L.F., Clark S., Nganga D., Brandariz K.L., Abbink P. (2011). International seroepidemiology of adenovirus serotypes 5, 26, 35, and 48 in pediatric and adult populations. Vaccine.

[53] Mast T.C., Kierstead L., Gupta S.B., Nikas A.A., Kallas E.G., Novitsky V., Mbewe B., Pitisuttithum P., Schechter M., Vardas E. (2010). International epidemiology of human pre-existing adenovirus (Ad) type-5, type-6, type-26 and type-36 neutralizing antibodies: Correlates of high Ad5 titers and implications for potential HIV vaccine trials. Vaccine.

[54] Seshidhar Reddy P., Ganesh S., Limbach M.P., Brann T., Pinkstaff A., Kaloss M., Kaleko M., Connelly S. (2003). Development of adenovirus serotype 35 as a gene transfer vector. Virology.

[55] Bru T., Salinas S., Kremer E.J. (2010). An update on canine adenovirus type 2 and its vectors. Viruses.

[56] McCoy K., Tatsis N., Korioth-Schmitz B., Lasaro M.O., Hensley S.E., Lin S.-W., Li Y., Giles-Davis W., Cun A., Zhou D. (2007). Effect of preexisting immunity to adenovirus human serotype 5 antigens on the immune responses of nonhuman primates to vaccine regimens based on human- or chimpanzee-derived adenovirus vectors. J. Virol..

[57] Stoff-Khalili M.A., Rivera A.A., Glasgow J.N., Le L.P., Stoff A., Everts M., Tsuruta Y., Kawakami Y., Bauerschmitz G.J., Mathis J.M. (2005). A human adenoviral vector with a chimeric fiber from canine adenovirus type 1 results in novel expanded tropism for cancer gene therapy. Gene Ther..

[58] Abbink P., Lemckert A.A.C., Ewald B.A., Lynch D.M., Denholtz M., Smits S., Holterman L., Damen I., Vogels R., Thorner A.R. (2007). Comparative seroprevalence and immunogenicity of six rare serotype recombinant adenovirus vaccine vectors from subgroups B and D. J. Virol..

[59] De Gruijl T.D., Ophorst O.J.A.E., Goudsmit J., Verhaagh S., Lougheed S.M., Radosević K., Havenga M.J.E., Scheper R.J. (2006). Intradermal delivery of adenoviral type-35 vectors leads to high efficiency transduction of mature, CD8^+^ T cell-stimulating skin-emigrated dendritic cells. J. Immunol..

[60] Hangalapura B.N., Oosterhoff D., de Groot J., Boon L., Tüting T., van den Eertwegh A.J., Gerritsen W.R., van Beusechem V.W., Pereboev A., Curiel D.T. (2011). Potent antitumor immunity generated by a CD40-targeted adenoviral vaccine. Cancer Res..

[61] Bagaev A.V., Pichugin A.V., Lebedeva E.S., Lysenko A.A., Shmarov M.M., Logunov D.Y., Naroditsky B.S., Ataullakhanov R.I., Khaitov R.M., Gintsburg A.L. (2014). Regulation of the target protein (transgene) expression in the adenovirus vector using agonists of toll-like receptors. Acta Nat..

[62] Nielsen K.N., Steffensen M.A., Christensen J.P., Thomsen A.R. (2014). Priming of CD8 T cells by adenoviral vectors is critically dependent on B7 and dendritic cells but only partially dependent on CD28 ligation on CD8 T cells. J. Immunol..

[63] Zhang J., Wang Y., Wu Y., Ding Z.-Y., Luo X.-M., Zhong W.-N., Liu J., Xia X.-Y., Deng G.-H., Deng Y.-T. (2014). Mannan-modified adenovirus encoding VEGFR-2 as a vaccine to induce anti-tumor immunity. J. Cancer Res. Clin. Oncol..

[64] Liljenfeldt L., Yu D., Chen L., Essand M., Mangsbo S.M. (2014). A hexon and fiber-modified adenovirus expressing CD40L improves the antigen presentation capacity of dendritic cells. J. Immunother..

[65] Hangalapura B.N., Timares L., Oosterhoff D., Scheper R.J., Curiel D.T., de Gruijl T.D. (2012). CD40-targeted adenoviral cancer vaccines: The long and winding road to the clinic. J. Gene Med..

[66] Ophorst O.J.A.E., Kostense S., Goudsmit J., De Swart R.L., Verhaagh S., Zakhartchouk A., Van Meijer M., Sprangers M., Van Amerongen G., Yüksel S. (2004). An adenoviral type 5 vector carrying a type 35 fiber as a vaccine vehicle: DC targeting, cross neutralization, and immunogenicity. Vaccine.

[67] Sumida S.M., Truitt D.M., Lemckert A.A.C., Vogels R., Custers J.H.H.V., Addo M.M., Lockman S., Peter T., Peyerl F.W., Kishko M.G. (2005). Neutralizing antibodies to adenovirus serotype 5 vaccine vectors are directed primarily against the adenovirus hexon protein. J. Immunol..

[68] Roberts D.M., Nanda A., Havenga M.J.E., Abbink P., Lynch D.M., Ewald B.A., Liu J., Thorner A.R., Swanson P.E., Gorgone D.A. (2006). Hexon-chimaeric adenovirus serotype 5 vectors circumvent pre-existing anti-vector immunity. Nature.

[69] Baden L.R., Walsh S.R., Seaman M.S., Johnson J.A., Tucker R.P., Kleinjan J.A., Gothing J.A., Engelson B.A., Carey B.R., Oza A. (2014). First-in-human evaluation of a hexon chimeric adenovirus vector expressing HIV-1 Env (IPCAVD 002). J. Infect. Dis..

[70] Gu L., Icyuz M., Krendelchtchikova V., Krendelchtchikov A., Johnston A.E., Matthews Q.L. (2016). Development of an Ad5H3 Chimera Using the “Antigen Capsid-Incorporation” Strategy for an Alternative Vaccination Approach. Open Virol. J..

[71] Sakurai F., Kawabata K., Mizuguchi H. (2007). Adenovirus vectors composed of subgroup B adenoviruses. Curr. Gene Ther..

[72] Liu M.A. (2010). Immunologic basis of vaccine vectors. Immunity.

[73] Wang H., Li Z.-Y., Liu Y., Persson J., Beyer I., ller T.M.O., Koyuncu D., Drescher M.R., Strauss R., Zhang X.-B. (2010). Desmoglein 2 is a receptor for adenovirus serotypes 3, 7, 11 and 14. Nat. Med..

[74] Elliott B., Cook M.G., John R.J., Powell B.W.E.M., Pandha H., Dalgleish A.G. (2004). Successful live cell harvest from bisected sentinel lymph nodes research report. J. Immunol. Methods.

[75] Kanerva A., Nokisalmi P., Diaconu I., Koski A., Cerullo V., Liikanen I., Tähtinen S., Oksanen M., Heiskanen R., Pesonen S. (2013). Antiviral and antitumor T-cell immunity in patients treated with GM-CSF-coding oncolytic adenovirus. Clin. Cancer Res..

[76] Zafar S., Parviainen S., Siurala M., Hemminki O., Havunen R., Tähtinen S., Bramante S., Vassilev L., Wang H., Lieber A. (2017). Intravenously usable fully serotype 3 oncolytic adenovirus coding for CD40L as an enabler of dendritic cell therapy. Oncoimmunology.

